# VEGF-A isoforms program differential VEGFR2 signal transduction, trafficking and proteolysis

**DOI:** 10.1242/bio.017434

**Published:** 2016-04-04

**Authors:** Gareth W. Fearnley, Gina A. Smith, Izma Abdul-Zani, Nadira Yuldasheva, Nadeem A. Mughal, Shervanthi Homer-Vanniasinkam, Mark T. Kearney, Ian C. Zachary, Darren C. Tomlinson, Michael A. Harrison, Stephen B. Wheatcroft, Sreenivasan Ponnambalam

**Affiliations:** 1 Endothelial Cell Biology Unit, School of Molecular and Cellular Biology, University of Leeds, Leeds LS2 9JT, UK; 2 Leeds Institute of Cardiovascular Metabolism and Medicine, LIGHT Laboratories, University of Leeds, Leeds LS2 9JT, UK; 3 Leeds Vascular Institute, Leeds General Infirmary, Great George Street, Leeds LS1 3EX, UK; 4 Centre for Cardiovascular Biology and Medicine, Division of Medicine, University College London, London WC1E 6BT, UK; 5 Biomedical Health Research Centre, Astbury Building, University of Leeds, Leeds LS2 9JT, UK; 6 School of Biomedical Sciences, University of Leeds, Leeds LS2 9JT, UK

**Keywords:** VEGF-A, VEGFR2, Endothelial, Trafficking

## Abstract

Vascular endothelial growth factor A (VEGF-A) binding to the receptor tyrosine kinase VEGFR2 triggers multiple signal transduction pathways, which regulate endothelial cell responses that control vascular development. Multiple isoforms of VEGF-A can elicit differential signal transduction and endothelial responses. However, it is unclear how such cellular responses are controlled by isoform-specific VEGF-A–VEGFR2 complexes. Increasingly, there is the realization that the membrane trafficking of receptor–ligand complexes influences signal transduction and protein turnover. By building on these concepts, our study shows for the first time that three different VEGF-A isoforms (VEGF-A_165_, VEGF-A_121_ and VEGF-A_145_) promote distinct patterns of VEGFR2 endocytosis for delivery into early endosomes. This differential VEGFR2 endocytosis and trafficking is linked to VEGF-A isoform-specific signal transduction events. Disruption of clathrin-dependent endocytosis blocked VEGF-A isoform-specific VEGFR2 activation, signal transduction and caused substantial depletion in membrane-bound VEGFR1 and VEGFR2 levels. Furthermore, such VEGF-A isoforms promoted differential patterns of VEGFR2 ubiquitylation, proteolysis and terminal degradation. Our study now provides novel insights into how different VEGF-A isoforms can bind the same receptor tyrosine kinase and elicit diverse cellular outcomes.

## Abstract (precis)

**Summary:** We show that vascular endothelial growth factor (VEGF-A) isoforms program differential tyrosine phosphorylation, ubiquitylation, endocytosis and proteolysis of the receptor tyrosine kinase, VEGFR2.

## INTRODUCTION

Vascular endothelial growth factor A (VEGF-A) is a soluble ligand that is essential for mammalian development and function (Carmeliet et al., 1996; Ferrara et al., 1996; Koch et al., 2011). VEGF ligands bind to a receptor tyrosine kinase (RTK) subfamily termed vascular endothelial growth factor receptors (VEGFR1, 2 and 3), which regulate many aspects of vascular and lymphatic development (Koch et al., 2011; Smith et al., 2015). The VEGF family member VEGF-A binds a major receptor and membrane glycoprotein (VEGFR2) expressed on endothelial cells and such interactions facilitate signal transduction events that control different aspects of vascular physiology. This VEGFR2 RTK is a major regulator of new blood vessel sprouting i.e. angiogenesis (Carmeliet, 2005; Koch and Claesson-Welsh, 2012).

Human VEGF-A is encoded by the *VEGFA* gene on locus 6p21.3 and contains at least eight exons and seven introns. The *VEGFA* primary RNA transcript undergoes alternative splicing to produce seven pro- and one anti-angiogenic isoforms of VEGF-A (Harper and Bates, 2008). However, the reasons for this VEGF-A isoform complexity and its conservation in mammalian species is unclear. In general, work in this field has focused on the VEGF-A_165_ isoform that is secreted by most animal cells and tissues; nonetheless, it is clear that other VEGF-A isoforms elicit important and distinct biological responses from endothelial cells (Harper and Bates, 2008; Smith et al., 2015). The VEGF-A_165_ isoform programs sequential steps in VEGFR2 tyrosine phosphorylation, ubiquitylation, trafficking and proteolysis (Bruns et al., 2010; Horowitz and Seerapu, 2012), linked to downstream signal transduction events that trigger pro-angiogenic outcomes such as cell proliferation, migration, tubulogenesis, vascular permeability and leukocyte recruitment (Fearnley et al., 2014a; Koch et al., 2011). Furthermore, VEGF-A isoforms differentially promote VEGFR2-dependent signal transduction and cellular responses (Fearnley et al., 2015, 2014a; Kawamura et al., 2008b; Pan et al., 2007). However, the underlying mechanism(s) by which VEGF-A isoforms act are still unclear, although VEGF-A isoform-specific binding is implicated in recruiting a co-receptor called neuropilin 1 (NRP1) (Ballmer-Hofer et al., 2011; Harper and Bates, 2008; Herzog et al., 2011; Kawamura et al., 2008a,b; Pan et al., 2007; Tillo et al., 2015). This membrane receptor can bind both VEGF-A_165_ and VEGF-A_121_ but only VEGF-A_165_ is believed to form a trimeric complex with VEGFR2 and NRP1 (Koch et al., 2011).

The role of membrane trafficking in regulating receptor-ligand function is becoming increasingly apparent (Bruns et al., 2010; Horowitz and Seerapu, 2012). For example, VEGF-A_165_-stimulated signal transduction requires co-ordination of VEGFR2 tyrosine kinase activation with residence at different locations within the endocytic pathway e.g. plasma membrane and endosomes (Gourlaouen et al., 2013; Jopling et al., 2009; Koch et al., 2014; Lanahan et al., 2013, 2010, 2014; Manickam et al., 2011; Nakayama et al., 2013; Yamada et al., 2014; Zhang et al., 2013). Plasma membrane VEGFR2 activation promotes recruitment of phospholipase Cγ1 thus stimulating phosphatidylinositol-4,5-bisphosphate (PIP_2_) hydrolysis to generate inositol-1,4,5-trisphosphate (IP_3_) and diacylglycerol (DAG): these molecules act as second messengers that promote cytosolic calcium ion flux and protein kinase C activation respectively (Meyer et al., 2003; Takahashi and Shibuya, 1997; Wong and Jin, 2005). However, VEGF-A-stimulated activation of the MAP kinase pathway is linked to VEGFR2 residence in early endosomes (Bruns et al., 2010; Jopling et al., 2009; Lampugnani et al., 2006; Lanahan et al., 2010).

An important question is whether VEGF-A isoforms have the capacity to differentially ‘program’ VEGFR2 trafficking and turnover that subsequently impacts on signal transduction and endothelial cell responses. By combining biochemical and cell biological approaches, our study finds that three different VEGF-A isoforms (VEGF-A_165_, VEGF-A_121_ and VEGF-A_145_) stimulate different patterns of VEGFR2 phosphorylation and internalization into early endosomes, which subsequently impact on downstream signal transduction events. Furthermore, such activated VEGFR2 polypeptides exhibit distinct patterns of ubiquitylation and proteolysis. Our work now shows that VEGF-A isoform-specific programming of VEGFR2 function is dependent on a combination of post-translation modifications linked to residence time within different compartments along the endocytic route.

## RESULTS

### VEGF-A isoforms promote differential signal transduction and endothelial responses

**Fig. 1.**
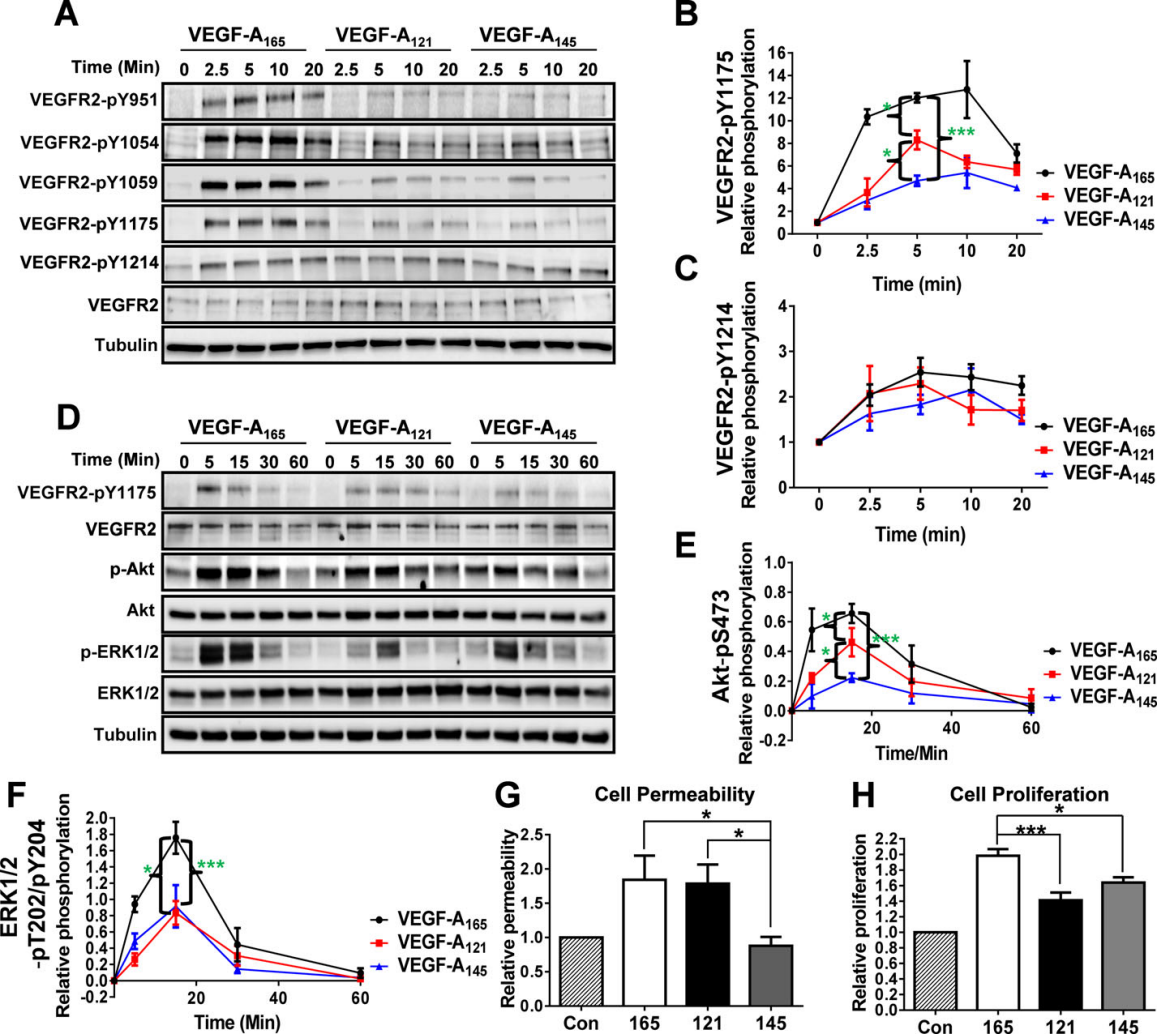
**VEGF-A isoforms promote differential VEGFR2 phosphorylation and downstream signal transduction.** (A) Endothelial cells were stimulated with either VEGF-A_165_, VEGF-A_121_ or VEGF-A_145_ (1.25 nM) for 2.5, 5, 10 or 20 min before lysis and processing for immunoblot analysis using site-specific phospho-antibodies against VEGFR2. (B,C) Quantification of VEGFR2-pY1175 (B) and VEGFR2-pY1214 (C) levels upon VEGF-A isoform stimulation. (D) Endothelial cells were stimulated with either VEGF-A_165_, VEGF-A_121_ or VEGF-A_145_ (1.25 nM) for 5, 15, 30 or 60 min before lysis and processing for immunoblot analysis of signal transduction. (E,F) Quantification of Akt-pS473 (E) and ERK1/2-pT202/pY204 (F) levels upon VEGF-A isoform stimulation. (G,H) Endothelial cells were seeded into cellular assays to assess endothelial cell permeability by measuring trans-endothelial electrical resistance (TEER) (G) or proliferation (H) upon control (Con) or VEGF-A_165_ (165), VEGF-A_121_ (121) or VEGF-A_145_ (145; 1.25 nM) stimulated conditions for 4 or 24 h respectively. Error bars indicate ±s.e.m. (*n*≥4). *P<0.05, **P<0.01, ***P<0.001.

VEGF-A binding to VEGFR2 activates multiple signal transduction pathways (e.g. ERK1/2, Akt and p38 MAPK) with evidence of VEGF-A isoform-specific cellular responses (Fearnley et al., 2015, 2014a; Kawamura et al., 2008b; Pan et al., 2007). Such intracellular signaling is dependent on VEGFR2 tyrosine phosphorylation on cytoplasmic residues such as Y951, Y1054, Y1059, Y1175 and Y1214 (Koch et al., 2011; Smith et al., 2016). To test the idea that VEGF-A isoforms trigger differential VEGFR2 activation, we monitored the presence of such VEGFR2 phosphotyrosine-epitopes in human umbilical vein endothelial cells (HUVECs) in response to stimulation with different VEGF-A isoforms (1.25 nM; 0-20 min) using immunoblot analysis (Fig. 1A). Quantification of these immunoblot data revealed that these three VEGF-A isoforms had differential capacities to promote the appearance of the VEGFR2-pY1175 epitope (Fig. 1B). However, another VEGFR2 phosphotyrosine epitope, pY1214, showed relatively similar profiles in response to VEGF-A isoform stimulation (Fig. 1C). Surprisingly, we discovered that significant levels of VEGFR2-pY1214 already existed in non-stimulated endothelial cells; furthermore, there was a relatively modest ∼2-fold rise in VEGFR2-pY1214 levels in response to any of the VEGF-A isoforms tested (Fig. 1A,C). Notably, the kinetics of VEGFR2-pY1214 levels displayed a more sustained profile (Fig. 1C), suggesting a more long-lived regulatory function.

Such findings raised the question whether other VEGFR2 phosphotyrosine epitopes exhibit distinct or different kinetic profiles. To evaluate this aspect, we monitored the appearance and kinetics of the VEGFR2-pY951, VEGFR2-pY1054 and VEGFR2-pY1059 epitopes using site-specific antibodies (Fig. 1A). All three VEGFR2 phosphotyrosine epitopes exhibited similar kinetics and profiles in response to stimulation with the VEGF-A_165_ isoform (Fig. S1A-C). Further analysis of these VEGFR2 phosphotyrosine epitopes in response to VEGF-A_121_ and VEGF-A_145_ isoform stimulation revealed ∼2-5-fold reduced signals with subtle profile differences (Fig. S1A-C). To examine signaling events downstream of VEGFR2-pY1175, we analyzed the phosphorylation and subsequent activation of Akt and ERK1/2 (Koch et al., 2011) (Fig. 1D). We found that the VEGF-A_165_ isoform promoted the highest increase in either Akt (Fig. 1E) or ERK1/2 (Fig. 1F) phosphorylation in comparison to the other VEGF-A isoforms. However, we noted that although VEGF-A_121_ was more effective (than VEGF-A_145_) in elevating VEGFR2-pY1175 (Fig. 1B) and Akt-pS473 (Fig. 1E) levels, both VEGF-A_121_ and VEGF-A_145_ caused relatively similar levels of phospho-ERK1/2 (Fig. 1F). One possible explanation for such effects is that VEGF-A isoform-specific programming of VEGFR2 phosphotyrosine epitopes is not the sole event(s) in controlling downstream signal transduction and subsequent cellular response(s).

In endothelial cells, VEGF-A-stimulated signal transduction through the Akt and ERK1/2 pathways is linked to increased cell permeability and proliferation respectively (Koch et al., 2011; Lal et al., 2001; Pedram et al., 1998; Six et al., 2002; Takahashi et al., 2001). To determine whether VEGF-A isoform-specific signal transduction differentially regulates such cellular responses, endothelial cells were subjected to VEGF-A isoform stimulation prior to the assessment of endothelial cell permeability and proliferation (Fig. 1G,H). VEGF-A_165_ and VEGF-A_121_ stimulation promoted ∼1.8-fold increase in endothelial monolayer permeability, and these effects were significantly higher than that observed upon VEGF-A_145_ stimulation (Fig. 1G). Analysis of endothelial cell proliferation upon VEGF-A_165_ isoform stimulation revealed an ∼2-fold increase, which was consistently higher (∼25-30%) than that observed upon either VEGF-A_121_ or VEGF-A_145_ stimulation (Fig. 1H). Additionally, VEGF-A_121_ and VEGF-A_145_ were comparable in their capacity to promote endothelial cell proliferation (Fig. 1H). We also compared the ability of these VEGF-A isoforms to stimulate endothelial cell migration, which revealed that all three VEGF-A isoforms caused ∼2-fold increase in cell migration with no isoform-specific effects (Fig. S2A,B). Other unique VEGF-A-stimulated cellular responses include the endothelial capacity to form tubules (tubulogenesis) or sprouts from arterial slices. Here, stimulation with the VEGF-A_165_ isoform was clearly the most effective at promoting endothelial tubulogenesis (Fig. S2C,D) and aortic sprouts (Fig. S2E,F) compared to the other two VEGF-A isoforms. Taken together, such data supports the idea that unique features encoded by each VEGF-A isoform enables the programming of specific patterns of VEGFR2 activation linked to downstream signal transduction events, which regulate endothelial cell responses.

### VEGF-A isoforms cause differential plasma membrane-to-endosome trafficking of VEGFR2

**Fig. 2.**
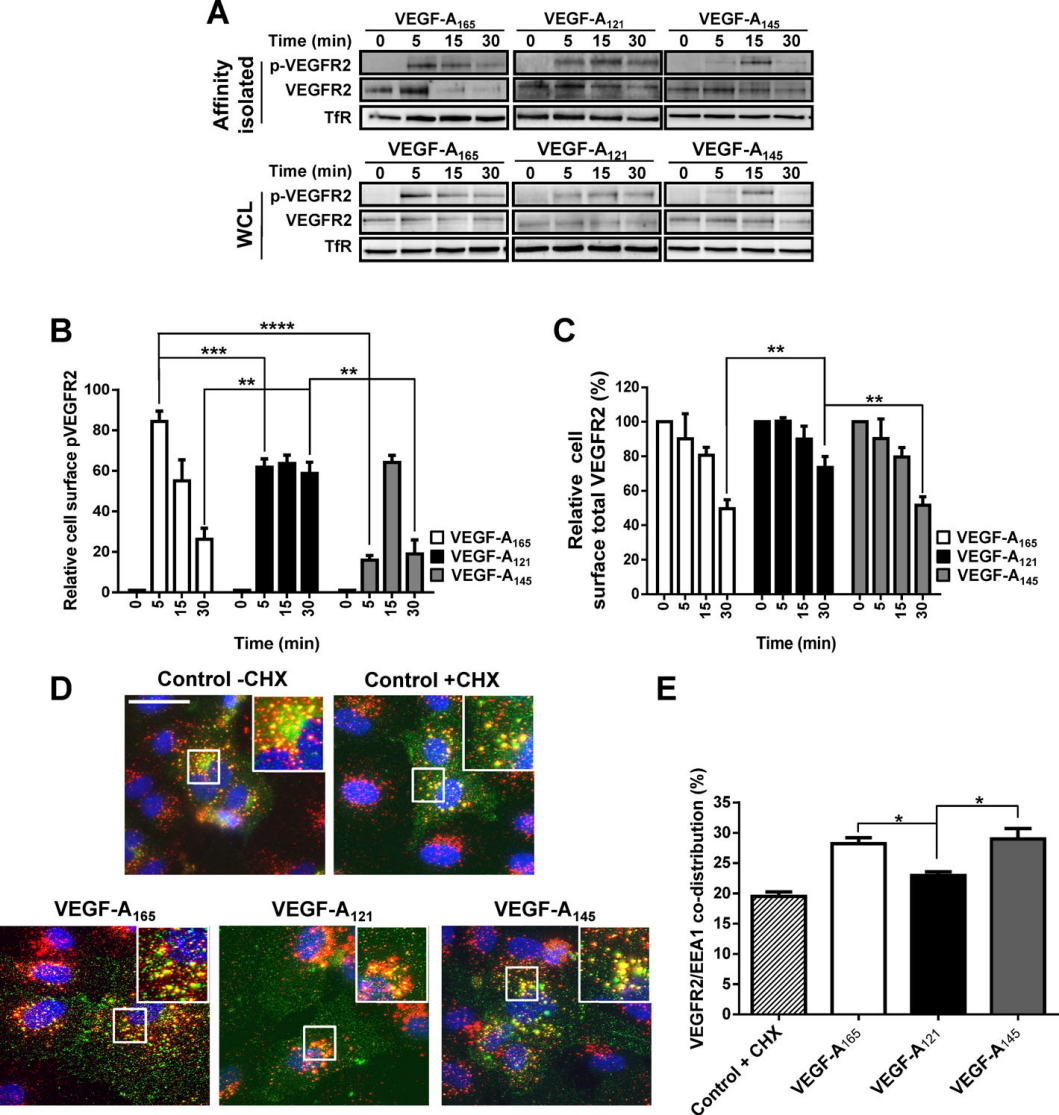
**VEGF-A isoforms promote differential ligand-stimulated VEGFR2 internalization.** (A) Endothelial cells were stimulated with either VEGF-A_165_, VEGF-A_121_ or VEGF-A_145_ (1.25 nM) for 5, 15 or 30 min before cell surface biotinylation, affinity isolation and immunoblot analysis of whole cell lysates (WCL) or biotinylated cell surface proteins (Affinity isolated). (B,C) Quantification of cell surface (B) activated VEGFR2-pY1175 or (C) mature total VEGFR2 levels upon VEGF-A isoform stimulation. Transferrin receptor (TfR) was used as a loading control. (D) Endothelial cells were pre-treated with cycloheximide (CHX; 2 μg/ml) for 2 h prior to VEGF-A isoform stimulation (1.25 nM) for 30 min. Endothelial cells were fixed and processed for immunofluorescence microscopy; VEGFR2 (green), EEA1 (red), nuclei (blue). Scale bar, 20 mm. (E) Quantification of VEGFR2/EEA1 co-distribution upon VEGF-A stimulation. Error bars indicate ±s.e.m. (*n*≥3). *P<0.05, **P<0.01, ***P<0.001, ****P<0.0001.

Recent studies have highlighted the importance of plasma membrane-to-endosome trafficking in regulating VEGFR2 activation and downstream signal transduction (Gaengel and Betsholtz, 2013; Gourlaouen et al., 2013; Lanahan et al., 2013, 2010; Nakayama et al., 2013). Our studies revealed that VEGF-A isoforms have unique properties to stimulate VEGFR2 activation and downstream signal transduction (Fig. 1); therefore, we hypothesized that different VEGF-A isoforms could differentially regulate VEGFR2 endocytosis. To test this idea, we assessed these VEGF-A isoforms for their effects on the pools of VEGFR2 located at the cell surface versus internal compartments using cell surface biotinylation, affinity enrichment and quantitative immunoblotting (Fig. 2A-C). Furthermore, we monitored the presence of VEGFR2-pY1175 within the biotinylated VEGFR2 pool at the cell surface (Fig. 2A). Quantification of these immunoblot data revealed that as expected, all three VEGF-A isoforms promoted a significant rise in cell surface VEGFR2-pY1175 levels, peaking at either 5 min (VEGF-A_165_ and VEGF-A_121_) or 15 min (VEGF-A_145_) respectively (Fig. 2B). Interestingly, cell surface VEGFR2-pY1175 levels were significantly decreased after 30 min of VEGF-A_165_ or VEGF-A_145_ stimulation (Fig. 2B). In contrast, VEGF-A_121_ caused a more long-lived pool of VEGFR2-pY1175 at the cell surface that persisted for at least 30 min (Fig. 2B). Both VEGF-A_165_ and VEGF-A_145_ produced similar kinetics of VEGFR2 endocytosis at the plasma membrane; however, VEGF-A_121_-stimulated internalization of VEGFR2 was clearly slower (Fig. 2C). These data suggest that these VEGF-A isoforms have differential abilities in not only promoting VEGFR2 activation but also causing plasma membrane-to-endosome trafficking.

To assess the localization of VEGFR2 in intracellular compartments in response to VEGF-A isoform stimulation, we used quantitative immunofluorescence microscopy to monitor VEGFR2 accumulation within early endosomes (Fig. 2D). By evaluating VEGFR2 co-distribution with an early endosome marker (EEA1), we discovered that either VEGF-A_165_ or VEGF-A_145_ caused a similar ∼30% increase in VEGFR2 accumulation within this compartment (Fig. 2E). In contrast, VEGF-A_121_ stimulation did not cause a significant increase in VEGFR2 co-distribution with EEA1 (Fig. 2E). Taken together, these data strongly support the view that VEGF-A isoforms have distinct properties in programming VEGFR2 plasma membrane-to-endosome trafficking.

### VEGF-A isoforms program differential VEGFR2 ubiquitylation and proteolysis

**Fig. 3.**
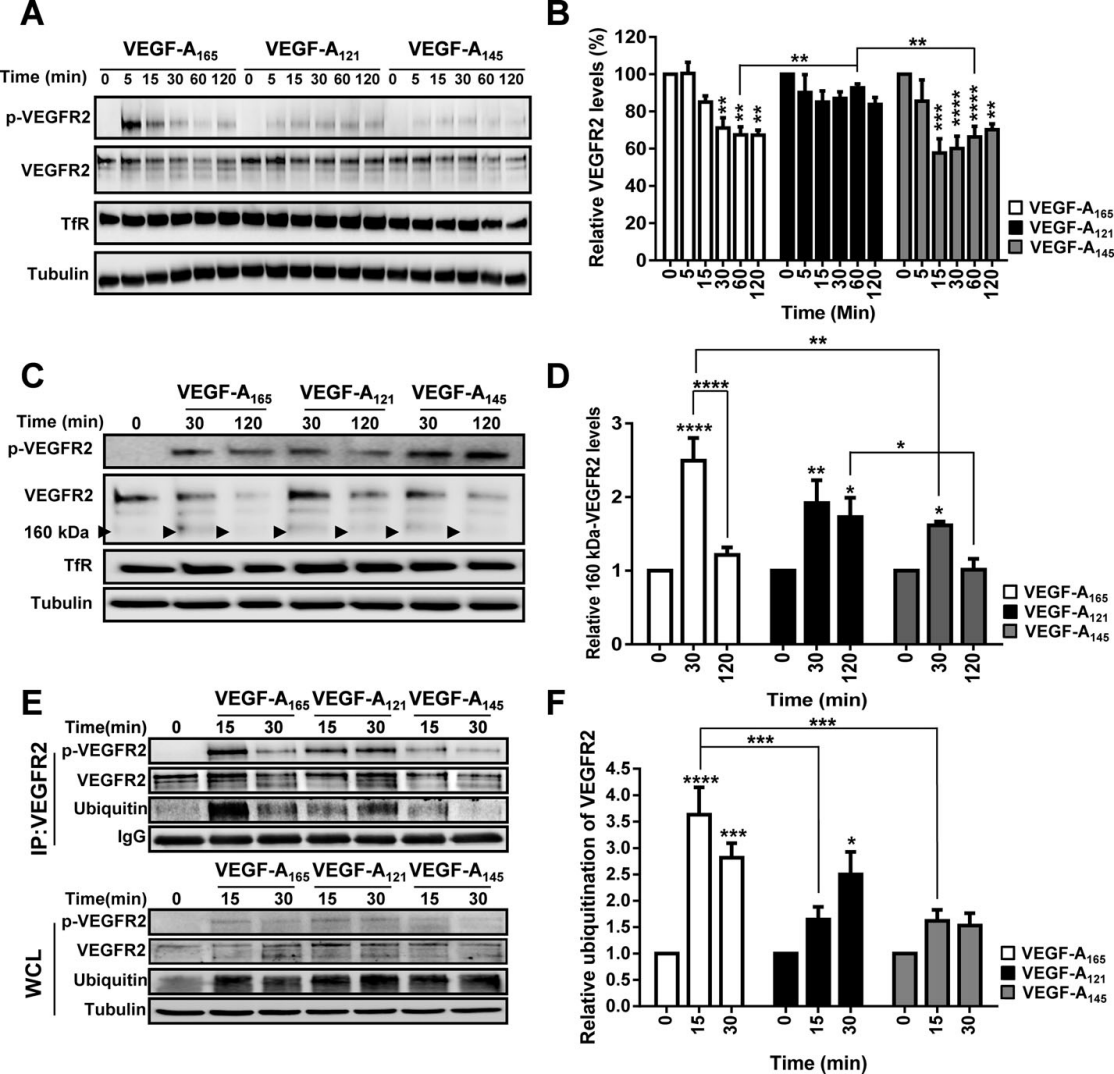
**VEGF-A isoform-specific regulation of ligand-stimulated VEGFR2 degradation, proteolysis and ubiquitylation.** (A) Endothelial cells were stimulated with either VEGF-A_165_, VEGF-A_121_ or VEGF-A_145_ (1.25 nM) for 5, 15, 30, 60 or 120 min before lysis and processing for immunoblot analysis to assess total VEGFR2 levels. (B) Quantification of total VEGFR2 levels upon VEGF-A isoform stimulation. (C) Endothelial cells were stimulated with either VEGF-A_165_, VEGF-A_121_ or VEGF-A_145_ (1.25 nM) for 30 or 120 min before lysis and processing for immunoblot analysis to assess VEGFR2 proteolysis. Black arrowheads denote ∼160 kDa proteolytic fragment. (D) Quantification of VEGFR2 160 kDa proteolytic fragment (arrowheads in C) levels upon VEGF-A isoform stimulation. (E) Endothelial cells were stimulated with either VEGF-A_165_, VEGF-A_121_ or VEGF-A_145_ (1.25 nM) for 15 or 30 min before being subjected to immunoprecipitation using an antibody against total VEGFR2 (IP:VEGFR2). Whole cell (WCL) or IP:VEGFR2 lysates were processed for immunoblot analysis to assess VEGFR2 ubiquitylation status using an antibody against poly-ubiquitin (FK2). Tubulin or IgG were used as loading controls for WCL or IP lysates respectively. (F) Quantification of VEGFR2 ubiquitylation status upon VEGF-A isoform stimulation. Error bars indicate ±s.e.m. (*n*≥3). *P<0.05, **P<0.01, ***P<0.001, ****P<0.0001.

VEGF-A binding to VEGFR2 triggers distinct patterns of ubiquitylation, proteolysis and clearance within the endosome-lysosome system (Bruns et al., 2010, 2012; Ewan et al., 2006; Smith et al., 2016). From our findings that VEGF-A isoforms had distinct properties in regulating VEGFR2 endocytosis at the plasma membrane, we predicted that VEGF-A-stimulated VEGFR2 degradation was isoform-specific. We confirmed that this was indeed true: VEGF-A_121_ showed negligible effects on VEGFR2 degradation (Fig. 3A,B); however, VEGF-A_165_ or VEGF-A_145_ showed a similar ∼30% reduction in total VEGFR2 levels after 120 min (Fig. 3B).

Previous studies have shown that VEGFR2 undergoes 26S proteasome-dependent proteolysis (∼30 min post-stimulation) resulting in the appearance of ∼160 kDa VEGFR2-derived proteolytic fragment (Bruns et al., 2010). In this context, one possibility was that VEGF-A isoforms programmed different patterns of VEGFR2 proteolysis. To test this idea, we subjected endothelial cells to VEGF-A isoform stimulation for 30 or 120 min and assessed VEGFR2 proteolysis by monitoring the presence of this 160 kDa proteolytic fragment using immunoblotting (arrowhead; Fig. 3C). Quantification of these data showed that all three VEGF-A isoforms significantly promoted VEGFR2 proteolysis, with a peak at ∼30 min post-stimulation (Fig. 3D). However, VEGF-A_165_ stimulation was the most effective at causing VEGFR2 proteolysis (∼2.5-fold increase) compared to VEGF-A_121_ or VEGF-A_145_ (Fig. 3D). Interestingly, VEGF-A_121_ stimulation also caused significant VEGFR2 proteolysis, which was higher than that observed upon VEGF-A_145_ stimulation (Fig. 3D). One conclusion drawn from these data is that a reduction in VEGFR2 endocytosis does not immediately correlate with a reduction in proteolysis in endosomes.

VEGF-A-stimulated VEGFR2 activation triggers increased receptor ubiquitylation, which is implicated in targeting VEGFR2 for proteolysis and degradation (Bruns et al., 2010; Ewan et al., 2006; Zhang et al., 2010). One possible explanation for the unique properties of each VEGF-A isoform to program distinct patterns of VEGFR2 proteolysis and degradation could be through programming specific patterns of VEGFR2 ubiquitylation. To test this idea, we subjected endothelial cells to different VEGF-A isoforms stimulation, immunoisolated VEGFR2 and probed these complexes for their ubiquitylation status via immunoblotting (Fig. 3E). We found that VEGF-A_165_ stimulation promoted maximal VEGFR2 ubiquitylation (Fig. 3E) corresponding to ∼3.5-fold increase (Fig. 3F). This ubiquitylation signal was significantly greater than that caused by either VEGF-A_121_ or VEGF-A_145_ stimulation (Fig. 3E,F). Interestingly, although VEGF-A_121_ stimulation caused significant VEGFR2 ubiquitylation with a different kinetic profile (Fig. 3F), VEGFR2 degradation was greatly reduced (Fig. 3B). Contrastingly, a further finding is that although VEGF-A_145_ promoted significant VEGFR2 degradation (Fig. 3B), it did not cause a corresponding increase in VEGFR2 ubiquitylation (Fig. 3E). Based on these data, we conclude that VEGFR2 ubiquitylation is not a prerequisite for degradation but could be required for proteasome-regulated VEGFR2 cleavage on endosomes.

### Disruption of clathrin-dependent VEGFR2 trafficking, results in the loss of isoform-specific signal transduction

**Fig. 4.**
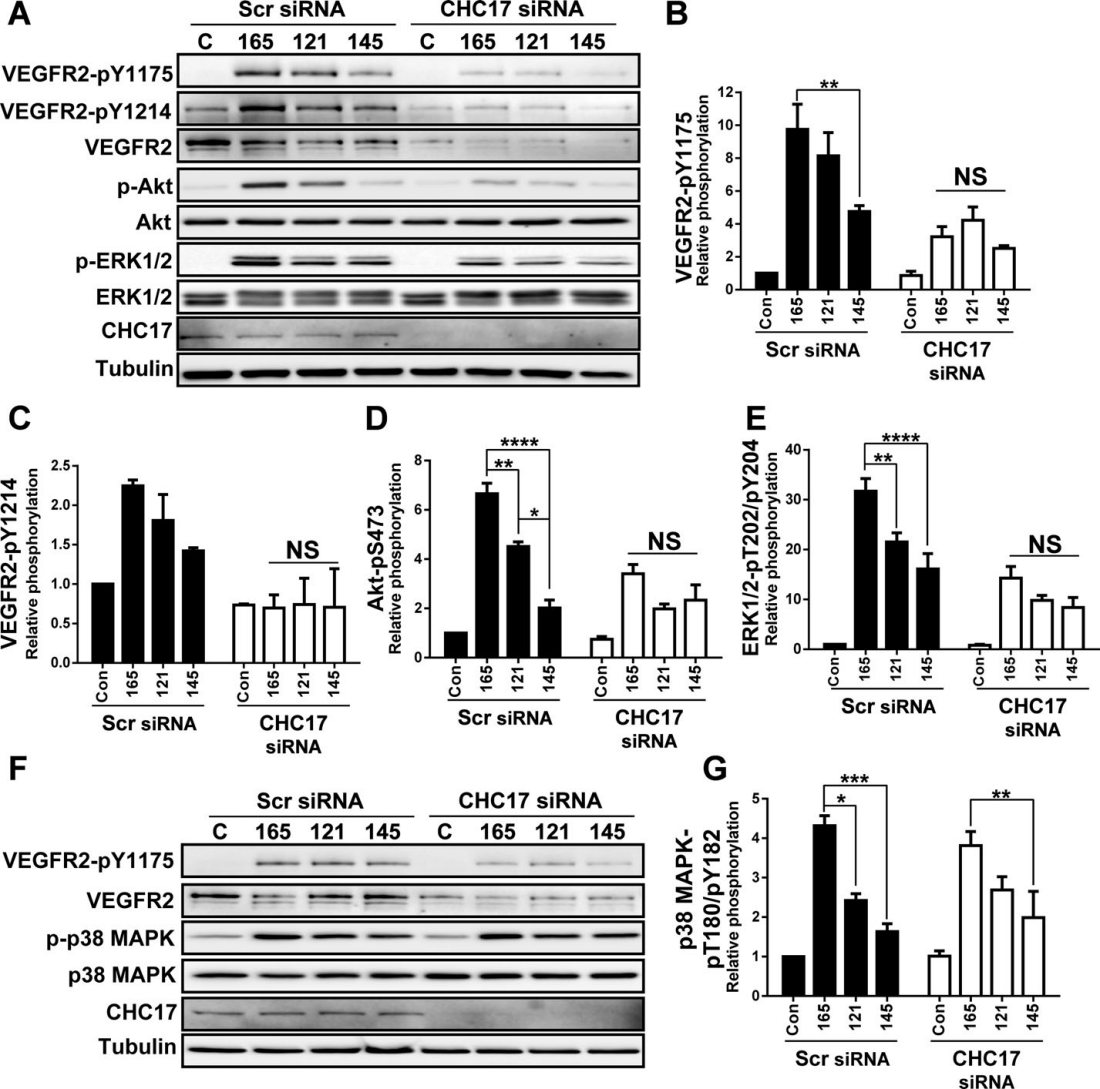
**Depletion of clathrin heavy chain disrupts VEGF-A isoform-specific programing of Akt and ERK1/2, but not p38 MAPK activation.** (A) Endothelial cells were subjected to scrambled (Scr) or clathrin heavy chain (CHC17)-specific siRNA duplexes, were stimulated with VEGF-A_165_ (165), VEGF-A_121_ (121) or VEGF-A_145_ (145; 1.25 nM) for 0 or 15 min prior to cell lysis and processing for immunoblot analysis of signal transduction. (B-E) Quantification of VEGFR2-pY1175 (B) and VEGFR2-pY1214 (C), Akt-pS473 (D) and ERK1/2-pT202/pY204 (E) levels upon VEGF-A isoform stimulation. (F) Endothelial cells were subjected to scrambled (Scr) or clathrin heavy chain (CHC17)-specific siRNA duplexes, were stimulated with either VEGF-A_165_, VEGF-A_121_ or VEGF-A_145_ (1.25 nM) for 15 min prior to cell lysis and processing for immunoblotting. (G) Quantification of p38 MAPK-pT180/pY182 levels upon VEGF-A isoform stimulation. Error bars indicate ±s.e.m. (*n*=3). *P<0.05, **P<0.01, ***P<0.001, ****P<0.0001.

Clathrin is an important structural protein that regulates clathrin-dependent endocytosis at the plasma membrane (Robinson, 2015). This endocytic route is further accessed by VEGFR2 complexes upon VEGF-A stimulation (Bruns et al., 2010; Ewan et al., 2006; Lampugnani et al., 2006). We hypothesized that targeted disruption of clathrin-dependent endocytosis of VEGFR2 would perturb VEGF-A isoform-specific signal transduction. To test this idea, we depleted clathrin heavy chain (CHC17) levels in endothelial cells using duplex siRNAs directed at the mRNA. Such treatment caused a substantial reduction in CHC17 protein levels (Fig. 4A). Unexpectedly, depletion of CHC17 and perturbation of clathrin-dependent endocytosis caused a substantial (>2-fold) decrease in VEGFR2-pY1175 and VEGFR2-pY1214 levels caused by stimulation with any of the VEGF-A isoforms (Fig. 4A–C). This reduced activation was also evident upon monitoring VEGF-A isoform-mediated activation of either Akt (Fig. 4A,D) or ERK1/2 enzymes (Fig. 4A,E). One major consequence occurring upon CHC17 depletion is a decrease in steady-state VEGFR2 levels (Fig. 4A). Hence, one likely explanation for the reduction in VEGFR2 activation and downstream signal transduction upon clathrin depletion is a corresponding and substantial depletion of VEGFR2 levels.

One question that was raised is whether other VEGFR2-regulated signal transduction pathways are also affected by depletion of CHC17 and perturbation of clathrin-dependent endocytosis. Therefore, since different VEGF-A isoforms also cause differential activation of p38 MAPK (Fearnley et al., 2015, 2014a; Kawamura et al., 2008a), we analyzed effects of CHC17 depletion on this signal transduction pathway (Fig. 4F). Surprisingly and in contrast to previous findings, CHC17 depletion did not significantly affect the activation of the p38 MAPK pathway (Fig. 4F). Quantification of these data showed no significant differences in VEGF-A isoform-specific p38 MAPK activation between control or CHC-17-depleted conditions (Fig. 4G). Notably, the relative magnitude of VEGF-A isoform-specific p38 MAPK activation was also retained (Fig. 4G), in spite of the substantial reduction in VEGFR2 levels upon CHC17 depletion (Fig. 4F). One conclusion is that clathrin-dependent endocytosis regulates VEGF-A isoform-specific VEGFR2-mediated turnover and signal transduction via Akt and ERK1/2 signal transduction pathways; however, VEGF-A-stimulated p38 MAPK signal transduction likely involves a VEGFR2 pool linked to a different membrane trafficking pathway.

**Fig. 5.**
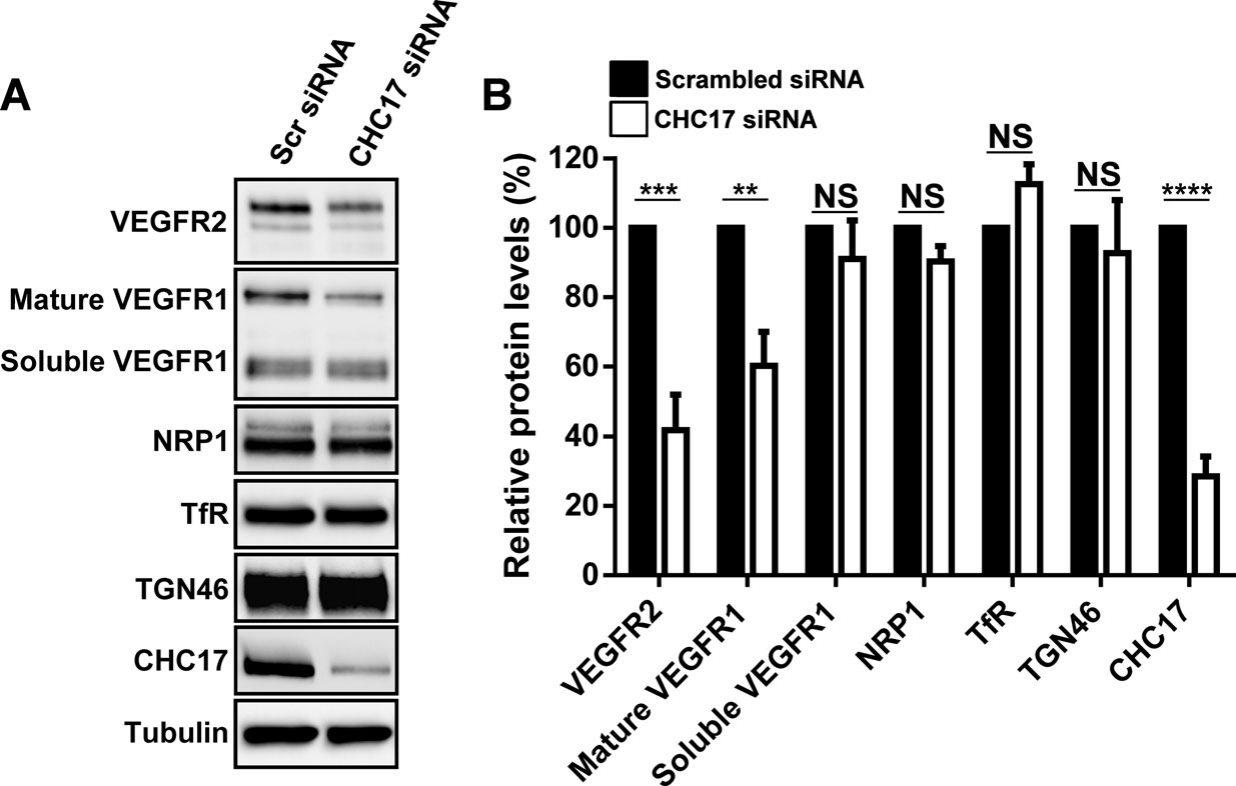
**Depletion of clathrin heavy chain reduces basal VEGFR2 and mature VEGFR1 levels.** (A) Endothelial cells were subjected to scrambled (Scr) or clathrin heavy chain (CHC17)-specific siRNA duplexes, prior to cell lysis and processing for immunoblot analysis. (B) Quantification of basal VEGFR2, VEGFR1, NRP1, transferrin receptor (TfR), TGN46 and CHC17 levels upon depletion of clathrin heavy chain (CHC17). Error bars indicate ±s.e.m. (*n*=3). **P<0.01, ***P<0.001, ****P<0.0001.

To evaluate the effects of perturbation of clathrin-dependent endocytosis on the turnover of different membrane proteins, we depleted clathrin heavy chain (CHC17) and examined the levels of VEGFR2, VEGFR1 and NRP1 using immunoblotting (Fig. 5A). As controls, we evaluated the turnover of the endosome-associated transferrin receptor (TfR) and the *trans*-Golgi network marker (TGN46) which are known to undergo recognition by clathrin-associated machinery, endocytosis and delivery to endosomes (Banting et al., 1998; Huang et al., 2004; Murphy et al., 2008; Owen and Evans, 1998). Quantification of these data showed that CHC17 depletion (∼70% reduction) caused ∼60% reduction in VEGFR2 and ∼40% reduction in VEGFR1 levels (Fig. 5B). Interestingly, although CHC17 depletion caused a reduction in membrane VEGFR1 levels, soluble VEGFR1 levels were not significantly affected (Fig. 5A,B). Furthermore, depletion of CHC17 and perturbation of clathrin-dependent endocytosis did not affect the turnover of TfR, NRP1 or TGN46 (Fig. 5A,B). One conclusion from these findings is that clathrin-dependent endocytosis regulates the turnover of mature membrane-bound VEGFR2 and VEGFR1 proteins. However, the turnover of other membrane proteins such as NRP1, TfR and TGN46 are not affected by blocking clathrin-dependent endocytosis.

## DISCUSSION

**Fig. 6.**
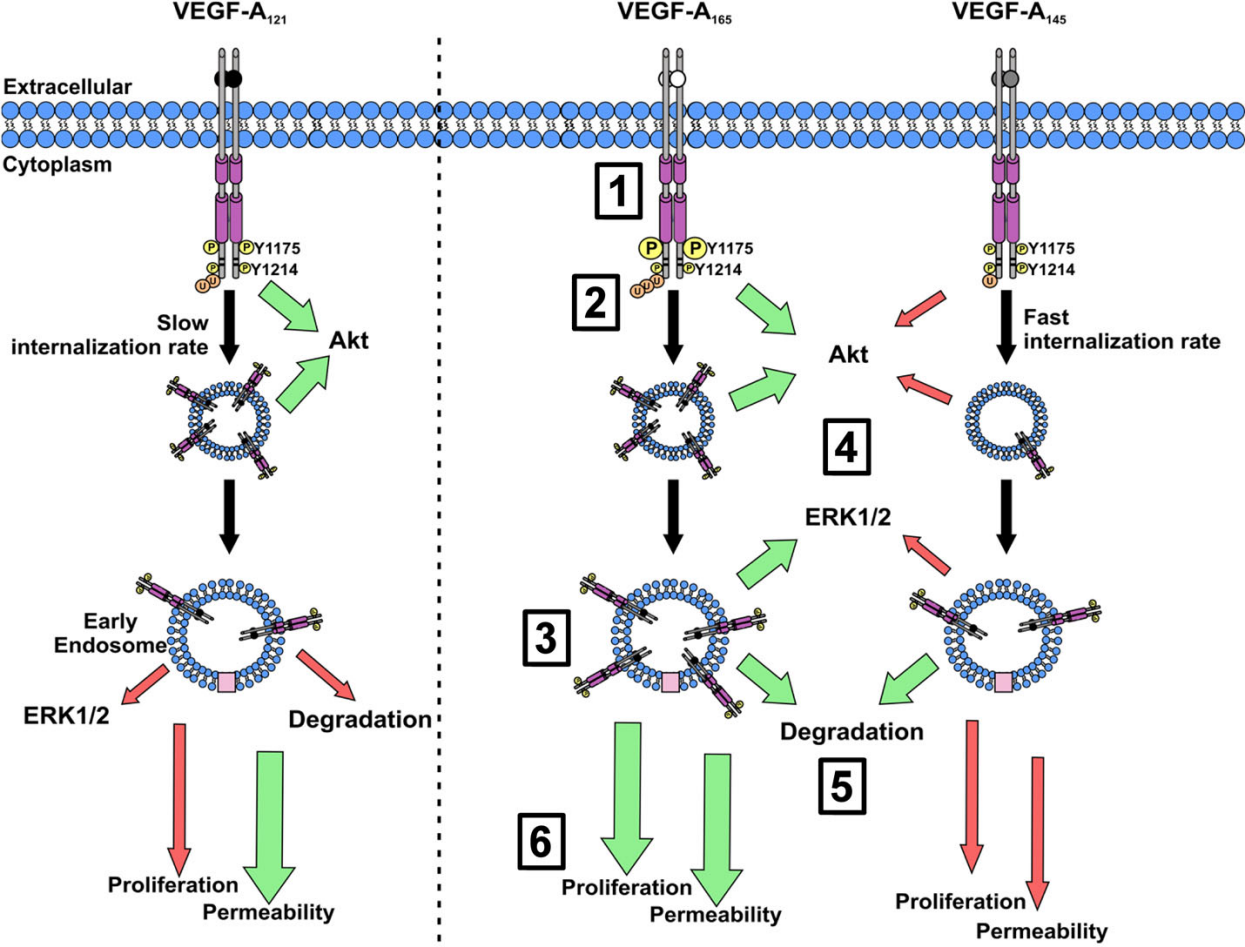
**Schematic depicting VEGF-A isoform-specific VEGFR2 trafficking and downstream signaling transduction.** Upon ligand binding (1) VEGFR2 undergoes dimerization and either differential (Y1175) or comparable (Y1214) trans-autophosphorylation of specific tyrosine residues, depending on the VEGF-A isoform used. (2) This results in distinct levels of receptor ubiquitylation (3) and internalization into EEA1-positive early endosomes. (4) Differential levels of VEGF-A isoform-stimulated VEGFR2 internalization impacts on Akt and ERK1/2 activation in combination with VEGFR2-Y1175 phosphorylation. (5) From early endosomes VEGFR2 is trafficked into late-endosomes where it under goes VEGF-A isoform-specific proteolysis prior to lysosomal degradation. (6) VEGF-A isoform-specific VEGFR2 activation and receptor trafficking, mediates their individual capacities to regulate endothelial cell permeability, proliferation and blood vessel formation. Size and magnitude of arrow denotes magnitude of response; red, reduced; green, increased.

Our study shows, for the first time, that different VEGF-A isoforms have unique properties in programming VEGFR2 endocytosis, ubiquitylation and proteolysis. In our model, three different VEGF-A isoforms can bind with similar affinity to the extracellular domain of this RTK (VEGFR2) but differentially program the cytoplasmic domain to acquire post-translational modifications, leading to specific patterns of trafficking and proteolysis (Fig. 6). We now suggest that activated VEGFR2 signified by acquisition of the pY1175 epitope undergoes endocytosis and delivery to early endosomes; such trafficking is essential for VEGF-A isoform-specific activation of the Akt and ERK1/2 signal transduction pathways (Fig. 6). This type of signal transduction is an essential feature of how different VEGF-A isoforms regulate the endothelial response that is central to the control of vascular physiology (Fig. 6).

Key lines of evidence support our conclusion that VEGF-A isoforms differentially stimulate VEGFR2 endocytosis and delivery to early endosomes (Fig. 6). Ligand-dependent VEGFR2 endocytosis was elevated in endothelial cells stimulated with either VEGF-A_165_ or VEGF-A_145_. In contrast, VEGF-A_121_ displayed negligible effects in promoting VEGFR2 endocytosis. One possible explanation lies in VEGF-A isoform-specific recruitment of VEGF co-receptors such as the neuropilins (NRP1, NRP2), which are implicated in regulating VEGFR signal transduction and trafficking (Ballmer-Hofer et al., 2011; Fearnley et al., 2014a; Herzog et al., 2011; Lanahan et al., 2013; Pan et al., 2007; Zachary, 2014). It has been proposed that both VEGF-A_165_ and VEGF-A_121_ can bind NRP1; however, only VEGF-A_165_ is believed to form a heteromeric complex with VEGFR2 and NRP1 (Koch et al., 2011). Thus differential NRP1 recruitment into a VEGFR2–VEGF-A complex depending on the VEGF-A isoform involved could be a mechanism of programming signaling and trafficking outcomes.

VEGFR2 trafficking is an essential regulatory component of VEGF-A_165_-stimulated signaling events, with evidence for different signal transduction pathways associated with the plasma membrane and early endosomes (Gaengel and Betsholtz, 2013; Gourlaouen et al., 2013; Lanahan et al., 2013, 2010; Nakayama et al., 2013). Our study further extends such ideas as depletion of clathrin heavy chain (CHC17) and perturbation of clathrin-dependent endocytosis impaired Akt and ERK1/2 activation but did not affect the p38 MAPK pathway. Surprisingly, clathrin heavy chain depletion caused ∼60% reduction in steady-state VEGFR2 levels but an explanation for this remarkable effect is not clear. One possibility is that multiple plasma membrane-associated VEGFR2 pools are linked to different trafficking pathways that impact on VEGFR2 recycling and turnover. Here, perturbation of clathrin-dependent endocytosis increases VEGFR2 proteolysis and turnover: increased plasma membrane VEGFR2 accumulation could result with association with clathrin-independent endocytosis pathways in endothelial cells such as caveolae (Mukherjee et al., 2006) and micropinocytosis (Muro et al., 2004). In contrast, a separate VEGFR2 pool that exhibits different long-lived plasma membrane dynamics couples VEGF-A binding to signal transduction via the p38 MAPK pathway. VEGFR2 has been previously documented to be also associated with clathrin-independent endocytic routes (Labrecque et al., 2003). A logical conclusion is that VEGFR2 accumulation at the plasma membrane caused by a block in clathrin-dependent endocytosis causes increased VEGFR2 trafficking (and degradation) via an alternative route from the plasma membrane e.g. caveolae and/or macropinocytosis.

An important discovery in this study is the finding that different VEGF-A isoforms have the capacity to program distinct patterns of VEGFR2 signal transduction and turnover. The VEGF-A_145_ and VEGF-A_165_ isoforms have comparable properties in programming VEGFR2 degradation; however, VEGF-A_145_ exhibits drastically reduced capacity for promoting VEGFR2 tyrosine phosphorylation. One explanation for these differences is that VEGF-A_165_ binding to VEGFR2 subsequently promotes NRP1 recruitment, and this heteromeric complex displays enhanced VEGFR2 recycling (Ballmer-Hofer et al., 2011). Thus failure of VEGF-A_145_ to promote NRP1 recruitment results in the shuttling of the VEGFR2–VEGF-A_145_ complex from early endosomes towards late endosomes and ultimately lysosomes for terminal degradation. In this context, heparan sulfate proteoglycan is also postulated to act as a co-receptor that modulates VEGF-A interactions with VEGFR2 and subsequent functional outcomes (Cohen et al., 1995; Jakobsson et al., 2006; Xu et al., 2011). Heparin-binding domains are present within VEGF-A_145_ and VEGF-A_165_ whereas VEGF-A_121_ lacks this region. These facts raise the possibility that heparan sulfate proteoglycan recruitment to the VEGFR2–VEGF-A complex further modulates signal transduction, trafficking and proteolysis events.

VEGFR2 ubiquitylation, proteolysis and terminal degradation is strongly linked to endothelial cell responses in normal and disease states (Bruns et al., 2010; Ewan et al., 2006; Pasula et al., 2012; Shaik et al., 2012). However, our findings in this study now argue that VEGFR2 ubiquitylation is not essential for controlled degradation. In support of this idea, stimulation with VEGF-A_145_ promoted a substantial increase in VEGFR2 degradation comparable to that induced by VEGF-A_165_, despite it stimulating relatively low levels of VEGFR2 ubiquitylation. Therefore, we now revise current models and suggest a mechanism whereby VEGFR2 ubiquitylation is a prerequisite for proteasome-regulated VEGFR2 cleavage on endosomes rather than lysosome-mediated terminal degradation (Fig. 6). This proposed mechanism is further strengthened by a recent study, which shows that VEGFR2 de-ubiquitylation is functionally coupled to different proteolytic steps on early endosomes (Smith et al., 2016). The selective proteolytic cleavage of the activated and ubiquitylated VEGFR2 complex can modulate communication to the ERK1/2 and Akt signal transduction pathways (Bruns et al., 2010). Thus VEGF-A isoform-specific VEGFR2 proteolysis and/or terminal degradation could be a mechanism to program signal transduction downstream of VEGFR2 in order to control diverse cellular responses.

An important finding in this study is that VEGF-A isoforms cause differential phosphorylation of tyrosine residues within the VEGFR2 cytoplasmic domain. A commonly postulated model is that ligand-induced RTK dimerization enables one RTK polypeptide to trans-phosphorylate the other ‘partner’ within the complex (Endres et al., 2014; Kovacs et al., 2015; Lemmon and Schlessinger, 2010; Lemmon et al., 2014). This is also postulated to occur here with VEGF-A-stimulated VEGFR2 dimerization promoting trans-autophosphorylation of multiple tyrosine residues. We now find that different VEGF-A isoforms promote distinct patterns of phosphorylation on VEGFR2 cytoplasmic residues Y951, Y1054, Y1059 and Y1175. One unexpected finding is the discovery that a phosphotyrosine epitope, VEGFR2-pY1214, is already present at significant levels in resting or non-stimulated cells. In contrast to other VEGFR2 phosphotyrosine epitopes analyzed in this study, VEGF-A stimulation only caused a modest twofold increase in VEGFR2-pY1214 levels with no evident isoform-specific effects. One possible explanation is that the VEGFR2-pY1214 epitope has a regulatory function(s) in the quiescent RTK state. This idea is supported by transgenic mouse studies where the *VEGFR2-Y1175F* germline mutation causes embryonic lethality and resembles *VEGFA* (+/−) mice; however, the *VEGFR2-Y1214F* mutant mice are viable and fertile (Sakurai et al., 2005). Interestingly, the presence of the VEGFR2-pY1214 epitope is linked to p38 MAPK activation (Koch et al., 2011; Lamalice et al., 2006); however, this view can be challenged by the finding that the VEGF-A_165_, VEGF-A_121_ and VEGF-A_145_ isoforms all cause comparable VEGFR2-pY1214 levels but promote different levels of p38 MAPK activation. One explanation for this discrepancy is that differential NRP1 recruitment to the VEGFR2–VEGF-A complex is also linked to p38 MAPK activation and blood vessel sprouting (Kawamura et al., 2008a). Thus the VEGF-A_165_ isoform-specific recruitment of NRP1 could account for its capacity to promote increased p38 MAPK signal transduction. We conclude that the two VEGFR2 phosphotyrosine-epitopes, pY1175 and pY1214, have different functional roles within the VEGFR2 complex and act by integrating different aspects of signal transduction, post-translational modifications and trafficking.

An emerging view is based on VEGF-A isoforms having unique properties in programming VEGFR2 endocytosis, phosphorylation, ubiquitylation, proteolysis and terminal degradation in lysosomes (Fig. 6). We now provide an integrated mechanism to explain how different VEGF-A isoforms regulate endothelial cell responses such as cell permeability and proliferation. Our study now provides novel insights into how multiple VEGF-A isoforms bind to the same RTK, yet elicit diverse biochemical and membrane trafficking outcomes that influence the cellular response. A future challenge will be to identify specific cytoplasmic factors that regulate the differences in VEGF-A isoform-mediated VEGFR2 trafficking and turnover. Such findings will provide a platform towards new ways of manipulating endothelial cell function in health and disease.

## MATERIALS AND METHODS

### Antibodies and growth factors

Antibodies: goat-anti-VEGFR1 (#AF321), goat-anti-VEGFR2 (#AF357), rabbit-anti-phospho-VEGFR2-Y1214 (#AF1766) (R&D Systems, Minneapolis, MN, USA), rabbit-anti-Akt (#9272S), rabbit-anti-phospho-Akt (S473; #4060B), rabbit-anti-ERK1/2 (#9102S), mouse-anti-phospho-ERK1/2 (T202/Y204; #9106S), rabbit-anti-neuropilin 1 (NRP1; #3725S), rabbit-anti-phospho-VEGFR2-Y951 (#4991S), rabbit-anti-phospho-VEGFR2-Y1059 (#3817S), rabbit-anti-phospho-VEGFR2-Y1175 (#2478S; Cell Signaling Technology, Danvers, MA, USA), rabbit-anti-phospho-VEGFR2-Y1054 (Clone D1W; #04-894; Merck Millipore, Watford, UK), mouse-anti-α-tubulin (Clone DM1A; #T6199; Sigma Aldrich, Poole, UK), mouse-anti-PECAM-1 (CD31; #sc-65260; Santa Cruz Biotechnology, Dallas, TX, USA), mouse anti-ubiquitin (FK2; #14220; Caymen Chemical, MI, USA), mouse-anti-clathrin heavy chain antibody (X22; #ab2731; Abcam, Cambridge, UK), sheep-anti-TGN46 (#AHP500GT; AbD Serotec, Oxford, UK). Endothelial cell growth medium (ECGM) was from PromoCell (Heidelberg, Germany). Recombinant human VEGF-A_165_ was from Genentech Inc. (San Francisco, CA, USA), both VEGF-A_121_ and VEGF-A_145_ was from Promocell.

### Cell culture and immunoblotting analysis

Human umbilical vein endothelial cells (HUVECs) were characterized as previously described (Fearnley et al., 2014b; Howell et al., 2004). Cells were seeded into 6-well plates and cultured (for at least 24 h) in ECGM until ∼80% confluent, washed three times with PBS and starved in MCDB131+0.2% (w/v) BSA for 2-3 h. HUVECs were stimulated with 1.25 nM of VEGF-A for the desired time period. Cells were washed three times with ice-cold PBS and lysed in 2% (w/v) SDS in TBS containing 1 mM PMSF and protease inhibitor cocktail (Sigma-Aldrich, Poole, UK). Protein concentration was determined using the bicinchoninic acid (BCA) assay (Thermo Fisher, Loughborough, UK). 15-25 µg of protein lysate was subjected to SDS-PAGE before analysis by immunoblotting.

### BrdU incorporation cell proliferation assay

2.5×10^3^ endothelial cells were seeded per well of a 96-well plate and left to acclimatize in ECGM overnight. Media was aspirated and cells starved in MCDB131+0.2% BSA (w/v) for 2 h. Cells were stimulated with VEGF-A isoforms (1.25 nM) in 100 µl total volumes for 24 h. 10 µM BrdU was added per well 20 h post-stimulation. A cell proliferation ELISA (Roche Diagnostics, Mannheim, Germany) was preformed according to manufacturer's instructions. The color change was developed using 3,3′5,5′-tetramethylbenzidine solution and the reaction quenched with 1 M H_2_SO_4_. Absorbance was measured at 450 nm using a Tecan Sunrise multiwavelength 96-well plate reader (Mannedorf, Switzerland).

### Assessment of endothelial monolayer permeability using trans-endothelial electrical resistance (TEER)

Human endothelial cells were seeded at 5×10^4^ cells/well into a 0.4 µm pore size Transwell filter inserted into a 24-well plate (BD Biosciences, Oxford, UK) in ECGM and cultured until a confluent monolayer was formed. Transwells containing endothelial cells were then washed twice, transferred to a fresh well containing 500 µl MCDB131+0.2% (w/v) BSA and starved in 400 µl MCDB131+0.2% (w/v) BSA (added to the top each chamber) for 2 h. After 2 h (t=0 h) the trans-endothelial electrical resistance (TEER) across each monolayer was measured using a MILLICELL-ERS TEER machine (Merck Millipore). Following which, 100 µl of MCDB131+0.2% (w/v) BSA containing the desired VEGF-A isoform was added to the upper chamber. After a further 4 h (t=4 h) TEER across each monolayer was measured again and the relative increase in permeability (corresponding to a decrease in electrical resistance across the endothelial monolayer) was calculated.

### Cell migration assay

Endothelial cells were seeded at 3×10^4^ cells/well into a 8 µm pore size Transwell filter inserted into a 24-well plate (BD Biosciences) in MCDB131+0.2% (w/v) BSA. MCDB131+0.2% (w/v) BSA containing the desired concentration of VEGF-A was added to the lower chambers to stimulate cell migration. Cells were allowed to migrate for 24 h before being fixed and stained with 0.2% (w/v) crystal violet in 20% (v/v) methanol. Non-migrated cells were removed from the upper chamber using a moist cotton bud; chambers were rinsed using double-distilled water. Three to five random fields were imaged per Transwell filter and the average number of migratory cells calculated.

### Tubulogenesis assay

Primary human foreskin fibroblasts (Promocell) were cultured until confluent in 48-well plates in Q333 fibroblast growth media (PAA Laboratories, Pasching, Austria). 7500 endothelial cells were then seeded per well onto the fibroblasts monolayer in a 1:1 mixture of Q333 and ECGM and left to acclimatize for 24 h. Media was then aspirated and replaced with fresh ECGM±VEGF-A isoform (1.25 nM) as desired; media was replaced every 2-3 days for seven days. Co-cultures were then fixed in 200 µl 10% (v/v) formalin for 20 min and blocked in 5% (w/v) BSA for 30 min at room temperature. Co-cultures were then incubated with 1 µg/ml mouse anti-human PECAM-1 (CD31; Santa Cruz, USA) overnight at 4°C. Cells were washed three times with PBS before incubation with an anti-mouse Alexa Fluor 594 conjugate (Invitrogen) for 3 h at room temperature. Wells were then washed three times with PBS. Endothelial tubules were visualized by immunofluorescence microscopy using an EVOS-fl inverted digital microscope (Life Technologies, Paisley, UK). Five random fields were imaged per well. Both the number of branch points and the total tubule length was then quantified from each photographic field using the open source software AngioQuant (www.cs.tut.fi/sgn/csb/angioquant) and values averaged. For a more detailed method please see Fearnley et al., (2014b).

### Aortic ring assay

Protocol adapted from previous studies (Baker et al., 2012). All procedures involving animals and their tissues were carried out in accordance to UK Home Office regulations and guidance at room temperature unless otherwise stated. Briefly, male wild type C57BL/6 mice were sacrificed in accordance with UK Home Office regulations. The thoracic aorta was harvested from aortic arch to diaphragm. Fat and fascia were removed from the aorta by sharp dissection and the vessel sliced into 0.5 mm rings. Aortic rings were serum starved overnight at 37°C in 5 ml OptiMEM supplemented with penicillin-streptomycin. On ice, purified type 1 rat tail collagen (Merck Millipore) was diluted to 1 mg/ml with DMEM before adding 2 µl per ml of 5 M NaOH. 55 µl of this embedding matrix was pipetted per well into a 96 well plate and aortic ring submerged within. Plates were incubated at room temperature for 15 min before incubation at 37°C for 90 min. 150 µl OptiMEM containing 2.5% (v/v) FCS and penicillin-streptomycin was added per well with appropriate VEGF-A isoform (1.25 nM). Aortic rings were incubated at 37°C for 5 days with a media change on day 3. Wells were washed with 150 µl PBS containing 2 mM CaCl_2_ and 2 mM MgCl_2_ and fixed in 4% formalin for 30 min. The collagen was permeabilized with three 15 min washes with PBS buffer containing 2 mM MgCl_2_, 2 mM CaCl_2,_ 0.25% (v/v) Triton X-100. Rings were blocked in 30 µl 1% (w/v) BSA in PBLEC (PBS containing 100 µM MnCl_2_, 1% (v/v) Tween-20, 2 mM CaCl_2_, 2 mM MgCl_2_) for 30 min at 37°C. 2.5 µg BS1 lectin-FITC (Sigma-Aldrich, Poole, UK) in PBLEC was added per well and incubated overnight at 4°C. Wells were washed three times with 100 µl PBS containing 2 mM MgCl_2_, 2 mM CaCl_2_ and 0.25% (v/v) Triton X-100 before incubation for 2 h with 1 µg/ml DAPI (in PBLEC). Wells were washed three times with 100 µl PBS containing 0.1% (v/v) Triton X-100 and then with 100 µl sterile water. Aortic sprouts were imaged using an EVOS-fl inverted digital microscope. Number of initial sprouts (vascular sprouts emanating directly from the aortic ring) were counted and averaged.

### Cell surface biotinylation

Endothelial cells were stimulated (1.25 nM VEGF-A isoform in MCDB131+0.2% (w/v) BSA) before washing twice with ice-cold PBS and incubation with 0.5 mg/ml EZ-Link Sulfo-NHS-LC-Biotin (Thermo Fisher) in PBS containing 2 mM MgCl_2_ and 2 mM CaCl_2_ for 30 min at 4°C. Biotinylation was quenched by washing twice with ice-cold TBS followed by washing twice with ice-cold PBS. Cells were lysed in 500 µl RIPA buffer for 1 h at 4°C. Lysates were cleared by centrifugation at 16,000 ***g*** for 30 min at 4°C. Equivalent protein amounts were incubated with 35 μl neutravidin-agarose beads (Thermo Fisher) overnight at 4°C. Beads were pelleted by brief centrifugation, supernatant removed and beads washed four times with 500 μl ice-cold RIPA buffer. 50 μl of 2× SDS-PAGE sample buffer was added and proteins eluted via heating at 92°C for 10 min before SDS-PAGE and immunoblotting.

### Immunofluorescence analysis

For immunofluorescence analysis, endothelial cells were serum starved in MCBD131+0.2% (w/v) BSA for 2 h before being stimulated for 30 min with desired VEGF-A isoform (1.25 nM). Endothelial cells were fixed and processed as previously described (Bruns et al., 2010). Images were acquired either using a wide-field deconvolution microscope DeltaVision (Applied Precision Inc., Issaquah, USA). Relative VEGFR2 co-distribution was quantified using Image J (NIH, Bethesda, USA) as previously described (Bruns et al., 2010; Jopling et al., 2011).

### VEGFR2 ubiquitylation analysis

Endothelial cells were stimulated (1.25 nM VEGF-A isoform in MCDB131+0.2% (w/v) BSA; 2 wells per condition) before washing twice with ice-cold PBS and lysed in RIPA buffer (150 mM NaCl, 50 mM Tris-HCl pH 7.4, 0.1% (w/v) SDS, 0.5% (w/v) sodium deoxycholate, 2 mM EDTA, 1% (v/v) NP-40, 50 mM NaF) with freshly added 1 mM phenylmethylsulfonyl fluoride (PMSF) and 10 mM iodoacetamide, and incubated for 5 min on ice at 4°C. Lysates were cleared via centrifugation at 16,000 ***g*** for 30 min at 4°C. Equal concentrations of supernatant were incubated with VEGFR2 antibody for 2 h at 4°C. 35 µl of 50:50 Protein G-sepharose slurry (Millipore, Watford, UK) was added and incubated overnight at 4°C. Beads were pelleted by brief centrifugation, supernatant removed and beads washed four times with 500 µl ice-cold RIPA buffer. 50 μl of 2× SDS-PAGE sample buffer was added and proteins eluted by heating at 92°C for 10 min before SDS-PAGE and immunoblotting. VEGFR2 ubiquitylation was monitored using mouse anti-FK2 antibody which detects both poly- and mono-ubiquitylation.

### Lipid-based transfection of siRNA duplexes

Cells were transfected with siRNA duplexes using lipofectamine RNAiMAX (Invitrogen). Per well of a 6-well plate, 15 µl of 2 µM siRNA duplexes was added to 481 µl of serum/antibiotic-free OptiMEM (Invitrogen) and allowed to settle at room temperature for 5 min. 4 µl of lipofectamine was then added and the mixture was inverted briefly and incubated at room temperature for 20 min. HUVECs were seeded at 2.5×10^5^ cells/ml in a 1 ml volume of OptiMEM, followed by immediate dropwise addition of the siRNA/lipofectamine mixture. Cells were left at room temperature for 30 min before being returned to the incubator. After 6 h total incubation, media was replaced for ECGM. Cells were allowed to recover for 72 h prior to treatment or processing for analysis. Scrambled siRNA was purchased as a siGENOME SMARTpool from Dharmacon (GE Healthcare, Buckinghamshire, UK), clathrin heavy chain (CHC17) siRNA was from Ambion (Life Technologies, Paisley, UK).

Scrambled siRNA target sequences: 5′-UAGCGACUAAACACAUCAA-3′, 5′-UAAGGCUAUGAAGAGAUAC-3′, 5′-AUGUAUUGGCCUGUAUUAG-3′, 5′-AUGAACGUGAAUUGCUCAA-3′. CHC17 siRNA target sequence: 5′-GGGUGCCAGAUUAUCAAUU-3′.

### Statistical analysis

This was performed using a one-way analysis of variance (ANOVA) followed by Tukey's post-hoc test or two-way ANOVA followed by Bonferroni multiple comparison test using GraphPad Prism software (La Jolla, CA, USA). Significant differences between control and test groups were evaluated with *P* values less than 0.05 (*), 0.01 (**), 0.001 (***) and 0.0001 (****) indicated on the graphs. Error bars in graphs and histograms denote ±s.e.m. (standard error of mean).
